# Fabrication of Textile Waste Fibers Aerogels with Excellent Oil/Organic Solvent Adsorption and Thermal Properties

**DOI:** 10.3390/gels8100684

**Published:** 2022-10-21

**Authors:** Chunlei Dong, Yangzhao Hu, Yuxuan Zhu, Jiale Wang, Xuerui Jia, Jianbing Chen, Jingliang Li

**Affiliations:** 1 Research Centre for Non-Metallic Materials, Chizhou University, Chizhou 247000, China; 2 Institute for Frontier Materials, Deakin University, Geelong, VIC 3200, Australia

**Keywords:** recycled textile waste, aerogels, oil spill cleaning, textile waste fibers

## Abstract

In recent years, the treatment of textile waste has attracted more and more attention around the world. The reuse of textile waste can contribute to the reduction of carbon emissions and the sustainable development of the economy. Herein, we proposed a facile and cost-effective approach to fabricating aerogel by using textile waste fibers as the matrix and polyvinyl alcohol (PVA) and glutaraldehyde (GA) as crosslinking agents. After being modified with methyltrimethoxysilane (MTMS) via chemical vapor deposition, both the interior and exterior of the textile waste aerogels exhibit a hydrophobic property with a water contact angle of up to 136.9° ± 2.3°. A comprehensive investigation of the structure, thermal properties, mechanical properties and oil absorption capacity of this aerogel shows its potential for building insulation and oil spill cleanup. The textile waste fibers aerogels have low density and high porosity, good thermal stability and outstanding heat insulation properties (K_avg._ = 0.049–0.061 W/m·K). With a maximum oil absorption value of 26.9 ± 0.6 g/g and rapid and effective oil/water mixture separation, the aerogel exhibits competitive commercial application value.

## 1. Introduction

The textile industry is still one of the largest and most vibrant industries in the world [1]. The consumption of textile products has increased tremendously from 78 million tons to more than 103 million tons during the last decade. As the economic development, the trend will increase while the pollution made from the waste textile will also increase [2,3,4]. The average consumption of textiles per person has increased from 7 kg in 1992 to 13 kg in 2013. According to one forecast, about 148 million tons of waste textiles will be produced in 2030, and more than 150 million tons of waste clothing will be incinerated or landfilled in 2050 [5]. The main components of textile wastes are polyester and cotton, which mainly include three categories, namely clothing, household materials and industrial textiles, which are mainly prepared by polyester and cotton [6,7,8]. Due to space constraints and leachate issues, the landfill method is banned in many regions and countries [9,10,11,12]. For example, European Union (EU) legislation has forbidden the landfill disposal of organic materials, including textile wastes, since 2016. Additionally, the member states of the EU will be required to set up a separate collection for discarded textiles by 2025. Incineration can reduce the number of textile wastes in a short period of time, while the combustion process of synthetic textiles will produce toxic chemicals (such as benzene derivatives and polycyclic aromatic hydrocarbons (PAHs)) and emit a large amount of greenhouse gas carbon dioxide [13,14].

Therefore, an environmentally benign disposal process to upcycle and recycle textile wastes is necessarily required to alleviate potential health, fossil energy and environmental issues. Textile waste materials have applications in the production of ethanol [15], glucose [11], nanocellulose and cellulose nanocrystals, microcrystalline cellulose [16], biogas [17], thermal and sound insulation materials [18], cement and bricks [3,19,20] and polymer composites [21], and so forth. The traditional treatment method of textile waste has complicated procedures or low economic value. In order to change the situation, there is an urgent need for more products with high economic value, such as aerogels, to enhance the financial value of textile waste recycling.

Aerogel, as a new material composed of a solid framework structure and two different phases of gaseous medium, has the characteristics of a typical nanoporous network structure, high specific surface area, high porosity, low density, excellent thermal insulation, outstanding acoustic insulation properties, low dielectric constant, high adsorption and so on [22]. Moreover, due to the size effect, surface effect and macroscopic quantum tunneling effect caused by the nanoscale of the skeleton and pores, they have been widely used in many fields such as mechanics, thermal science and optics [23]. Aerogel made from cotton fibers and polyester fiber, the two main components of textile waste, have been explored. Through different preparation processes, cotton fiber or polyester fiber can be applied to develop aerogels with various properties, such as oil absorption [24,25,26,27], solar steam generation [28,29,30], sensor [31,32,33], electrochemical capacitors [34], catalysis [35], thermal insulation [36,37,38], and so on.

The application prospect for aerogel is broad. With the rapidly developing new energy automobile industry around the world, the global market for automotive sound insulation and heat insulation materials is expected to reach 3.2 billion USD by 2022. Thermal insulation energy-saving buildings have become a new trend. In addition, the market for absorbents used to absorb spilled oil is expected to reach 177.63 billion USD by 2025 [39]. Driven by environmental issues and potential market application, we successfully developed aerogels from textile wastes with a facile and cost-effective method. The hydrophobicity, absorption capacity, mechanical property, oil/water separation and thermal conductivity of textile waste aerogels have been comprehensively studied for the application of oil spill cleaning and heat insulation.

## 2. Results and Discussion

### 2.1. Morphologies and Structures of Aerogels

**Figure 1:**
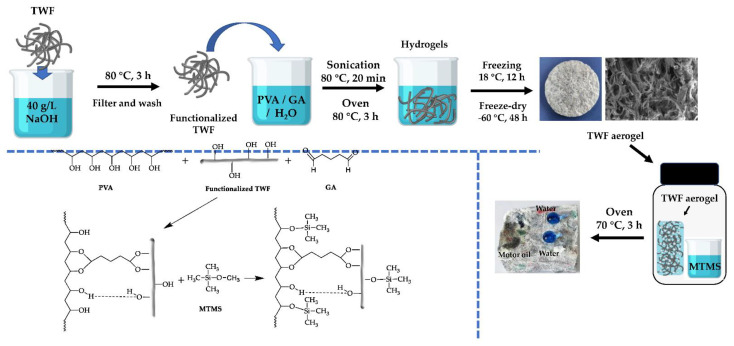
Schematic illustration of the preparation process of TWF aerogel and the possible reactions between PVA, TWF, GA and MTMS.

**Figure 2:**
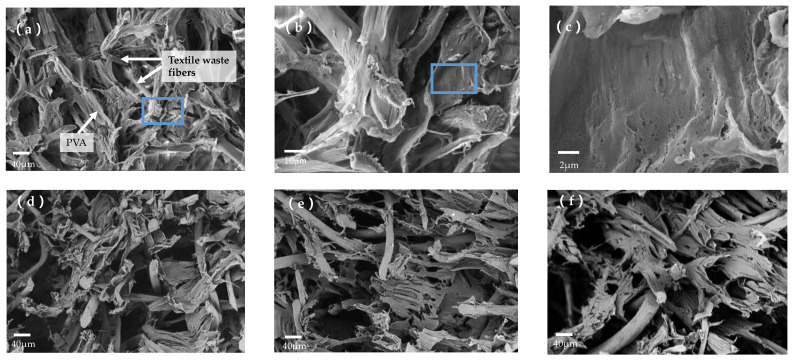
SEM images of TWF aerogels (**a**–**c**) TW01 with different magnifications; (**d**) TW02; (**e**) TW03; (**f**) TW04.

Figure 1 shows the preparation process of textile waste fibers (TWF) aerogel and the possible reactions between polyvinyl alcohol (PVA), TWF, glutaraldehyde (GA) and methyltrimethoxysilane (MTMS). The three-dimensional porous network of TWF aerogel was constructed through hydrogen and ester bonds formed between PVA and functionalized TWF and the use of GA as a cross-linker agent, which resulted in the generation of acetal bridges [26,40,41]. Figure 2 shows SEM images of the TWF aerogels with different magnifications. It can be observed that the internal structure of TWF aerogels has an open porous network structure with the uniform distribution of TWF in the matrix, indicating that TWF and PVA successfully self-assembled to form a three-dimensional porous network. As can be seen in Figure 2b, the enlarged blue frame of Figure 2a, the surface of fibers is rough and well bonded with PVA. In the TWF aerogel, the TWF and PVA networks are interlocked to form macropores, while small pores formed by freeze-drying are distributed in the PVA matrix gels-08-00684-tracked; these tiny pores can be observed in Figure 2c, which is an enlarged version of the blue frame in Figure 2b. To obtain a stable porous structure, the fibers are stuck together through PVA and GA. The TWF aerogel have a BET surface area in the 7.312.0 m^2^/g range, as shown in Table 1.

The waste fabrics are composed of polyester and cotton, with the hydroxyl group on the molecular chain [7,21,40]. TWF was pretreated with alkali can make its surface damaged with hydroxyl [42,43,44]. In the process of preparing the TWF gel solution, PVA can form hydrogen bonds with the hydroxyl groups on the surface-treated polymer fibers in the TWF [39,45,46]. According to previous reports [39,40,41,42,43,44,45,46], the addition of GA may react with hydroxyl groups of the various polymers in polyester, cotton and PVA chains to form acetal bridges of different chains, the possible reaction shown in Figure 1, which further enhances the interaction between TWF and PVA.

### 2.2. Hydrophobic Properties

**Figure 3:**
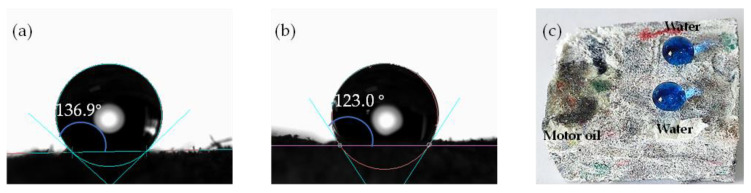
Water contact angles on (**a**) the external surface, (**b**) the cross-section of the MTMS-modified TWF aerogel and (**c**) digital pictures of water (dyed with methyl blue) and motor oil 5W-40 droplet on the surface of TWF aerogel.

The TWF aerogels obtained after freeze-drying were hydrophilic due to a large number of hydroxyl groups on the PVA and functionalized TWF. Therefore, for cleaning oil spill applications, it is necessary to modify the aerogel to transform its properties from hydrophilic to hydrophobic. To achieve this, MTMS was modified on the surface of the aerogel through a simple chemical vapor deposition process to generate hydrophobic silane groups (Figure 1 shows the possible reaction). For investigating the hydrophobicity of the coating aerogels effect, water contact angle measurement was performed on both the external and internal surfaces of aerogels. The aerogels without MTMS modified could immediately absorb water droplets during the test, resulting in no measurable contact angle. As shown in Figure 3a, the large contact angle of 136.9 ± 2.3° was measured on the external surface. To analyze the internal modified effect of aerogel, the sample was cut, and the water contact angle of 123.0 ± 2.5° was measured on the cross-section of the sample (Figure 3b), proving that the entire aerogel is hydrophobic. Therefore, it indicates that the porous network was successfully chemically modified by MTMS to make the entire aerogel hydrophobic. The water contact angle value of the external surface is higher than that of the cross-section, which may contribute to more opportunities for the external surface to contact MTMS and a higher degree of silanization. Figure 3c shows motor oil and blue water droplets (dyed with methyl blue) on the surfaces of TWF aerogel. Compared to the droplet of motor oil 5W-40, which quickly spreads and penetrates the TWF aerogel completely, the blue water droplets remain spherical on its surface. Consequently, the TWF aerogel exhibits oil/water separation capability due to its processing characteristics of wetting selectivity.

### 2.3. Thermal Properties of Aerogels

#### 2.3.1. Thermal Conductivity of Aerogels

**Figure 4:**
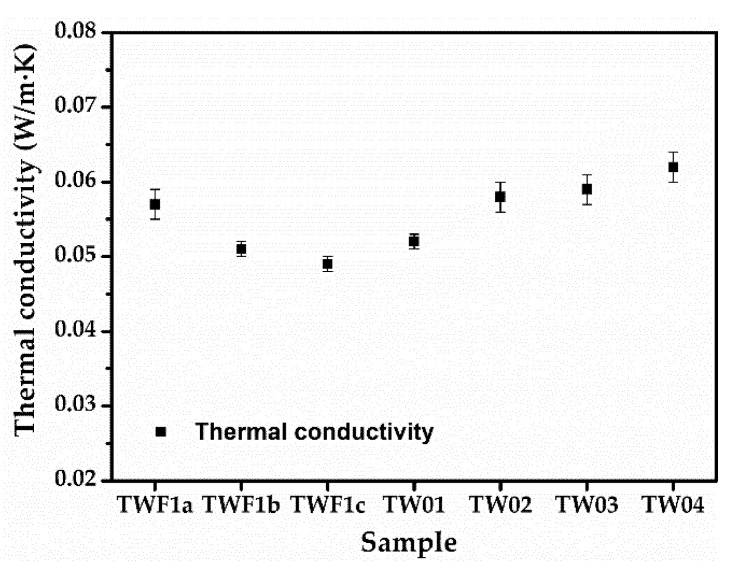
The thermal conductivity of TWF aerogels.

The thermal insulation properties of the TWF aerogels were characterized by thermal conductivity measurement at a room temperature of 25 °C and demonstrated in Figure 4 and Table 1. The TWF aerogels present excellent thermal insulation owing to low thermal conductivities (K_avg__._ = 0.049–0.062 W/m·K), which are comparable with conventional thermal insulation materials in residential and industrial buildings such as foams and wools (K_avg__._ = 0.030–0.055 W/m·K) [47]. The composition and morphology of the aerogel play a direct role in influencing and controlling the thermal conductivity of the aerogel. The air occupies most of the space in the porous structure of TWF aerogels, with a very low thermal conductivity (0.026 W/m·K) at ambient pressure and temperature [47]. The textile wastes used in this work mainly include polyester (PET) and cotton. PVA, PET and cotton have higher thermal conductivity, as shown in Table 2, and combine to form a framework that creates a complicated channel for heat to flow through. The thermal conductivity of PVA composite, polyester composite, cotton composite aerogel and textile waste fibers composite reported in recent years are displayed in Table 2. Although these values are close to or slightly larger than TWF aerogel, the raw material of TWF aerogel is cheaper, and the preparation procedure is more straightforward.

Figure 4 and Table 1 demonstrate that by keeping the amount of TWF constant, the thermal conductivity decreases with the increases in the ratio of TWF and PVA. Keeping the ratio of TWF and PVA unchanged, the thermal conductivity increases with the increase of TWF’s weight. In summary, the thermal conductivity increases with the increase of the total weight of the TWF and PVA. The thermal conductivity of TWF1c with the least weight and TW04 with the most weight is 0.049 ± 0.001 W/m·K and 0.061 ± 0.002 W/m·K, respectively. The changes in thermal conductivity follow the same trend as density, but in the opposite direction as porosity. It can be explained by the increase in the volume of the aerogel solid phase in the unit space, which leads to a decrease in the volume of internal air, thus increasing the thermal conductivity [46]. In addition, the increase of fibers in the TWF aerogel increases the heat transfer channels, which also causes an increase in thermal conductivity. Generally, TWF aerogels own ultra-low thermal conductivity and are prepared by textile wastes using environmentally friendly, low-cost and less toxic emissions methods, which are promising candidates for practical thermal insulation applications.

#### 2.3.2. Thermal Stability of Aerogels

**Figure 5:**
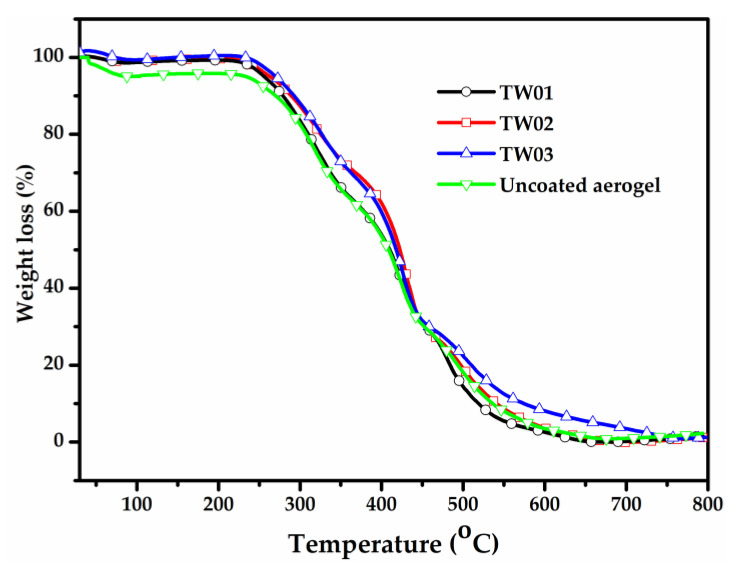
TGA curves of the TWF aerogels with different fiber concentrations.

In order to evaluate the thermal stability properties of TWF aerogels, TGA tests were measured and shown in Figure 5 and Table S1 with different concentrations. It can be observed that the mass change exhibit three phases by the temperature as follows: (1) 50–125 °C, (2) 220–500 °C and (3) 500–700 °C. Between 50 °C and 125 °C, the unmodified aerogel exhibits 4.5% weight loss, which likely is caused by the removal of the absorbed atmospheric moisture. The reason for this phenomenon may be that the materials contain many hydroxyl groups on PVA, which absorb water in the air after fabrication. On the other hand, negligible weight loss was shown on the aerogels with modified MTMS. At the next phase, between 220 and 450 °C, a weight loss of about 70 wt.% can be observed for all samples, likely due to the oxidation decomposition of the TWF and PVA. At the final thermal degradation between 500 °C and 700 °C, the weight of all samples decreases slightly, which possibly be caused by the oxidation of the charred residue.

### 2.4. Mechanical Properties of Aerogels

**Figure 6:**
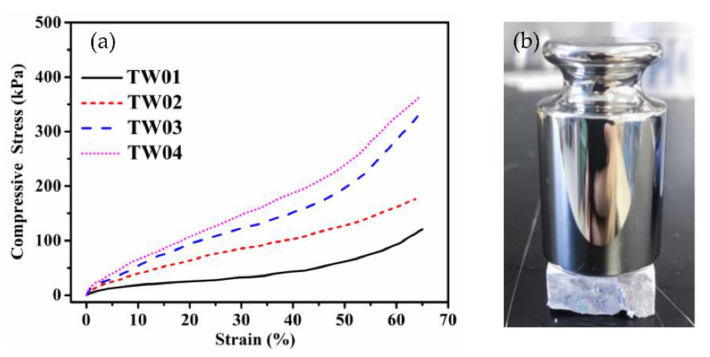
(**a**) The compressive stress-strain curves of the TWF aerogels with different fiber concentrations (**b**) A 200 g load on the TW01 aerogel for seven days.

The mechanical property of the TWF aerogel is a basic feature for practical applications. The mechanical strength property of the material and compression tests were performed. The compressive strain-stress curve and Young’s modulus of the TWF aerogels are presented in Figure 6a and Table S2, respectively. Figure 6b shows the TW04 was loading a 200 g weight on the sample for seven days. It was seen that no obvious shape change of the aerogel was found after the test durations. Additionally, there exists a noticeable trend: the compression resistance significantly increases when TWF concentration rises. With increasing from 2.0 wt.% to 5.0 wt.% of the TWF, Young’s modulus of the TWF aerogels is improved up 400% from 1.3 ± 0.16 kPa to 5.24 ± 0.22 kPa. It can be explained by a larger amount and more crosslinking sites of the TWF and PVA in the aerogels.

### 2.5. Absorption Capabilities of Aerogels

**Figure 7:**
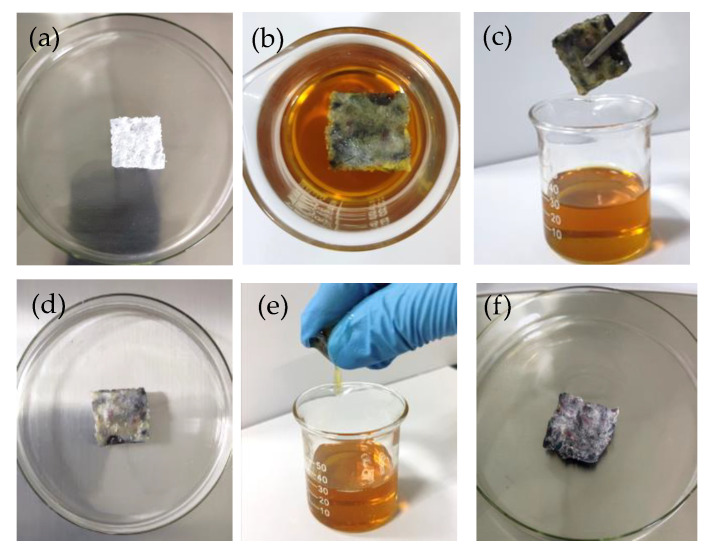
Motor oil absorption test with squeezing: (**a**) Weighing the aerogel sample before the test. (**b**) Absorption test with motor oil. (**c**) Draining sample after first absorption test. (**d**) Weighing sample after first absorption test. (**e**) Squeezing sample after the first absorption. (**f**) Weighing the sample after squeezing.

**Figure 8:**
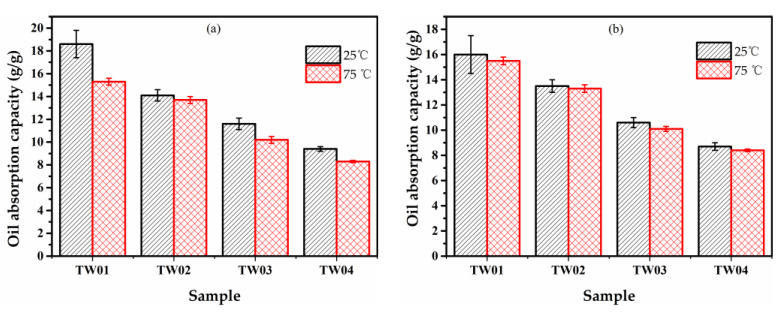
The absorption capabilities of motor oil (**a**) 5-W30 and (**b**) 5W-40.

The oil absorption capabilities of the TWF aerogels were investigated through the motor oil of 5W-30 and 5W-40. The process of motor oil absorption test with squeezing is exhibited in Figure 7. Figure 8 and Table S3 and show the first round of oil adsorption capacity. When the amount is kept to be TWF:PVA = 2 and increasing the fiber concentration from 2 to 5 wt.% at 25 °C or 75 °C, the measured 5W-30 and 5W-40 absorption capacities of the aerogels decreased, respectively. The maximum absorption capacity of 18.6 g/g is achieved with the 2 wt.% fiber aerogel for 5W-30 due to it having higher porosity and BET surface area. As exhibited in Table S3 and Figure 8, the maximum oil absorption capacity of TWF aerogels diminishes, when the temperature increases from 25 °C or 75 °C. This trend occurs in the absorption behavior of two oils on all aerogels. The absorption process of oils on adsorbent material is affected by compatibility between the oils and absorbents, capillary effect, van der Waals forces, pore morphology, diffusion effect and oil viscosity [57,58]. Temperature is considered to be the significant factor involving the viscosity and the diffusion rate of the oils penetrating into the interior of the aerogel. It can be observed in Table S4 that the viscosity of the oil decreases with increasing temperature, which facilitates oil penetration into the porous aerogel networks. On the other hand, the low viscosity reduces the amounts of the oil anchored in the porous structure of aerogel for the oils, having a negative effect on the total oil absorption of the aerogel. At 75 °C, the adsorption capacity of aerogels to the two kinds of motor oil is similar, due to the viscosity of the two oils being lower. At 25 °C, the amount of 5W-30 adsorbed is higher than 5W-40, probably due to 5W-30 having a lower viscosity than 5W-40, which is beneficial to diffusion into the porous structure. The amount of adsorption at 75 °C is lower than that at 25 °C caused by the viscosity of the oil is too low at 75 °C, which is not conducive to the anchoring of the oil inside the aerogel. The experimental results demonstrate that TWF aerogel with excellent oil absorption capacity could be the candidate material for oil spill cleaning.

**Figure 9:**
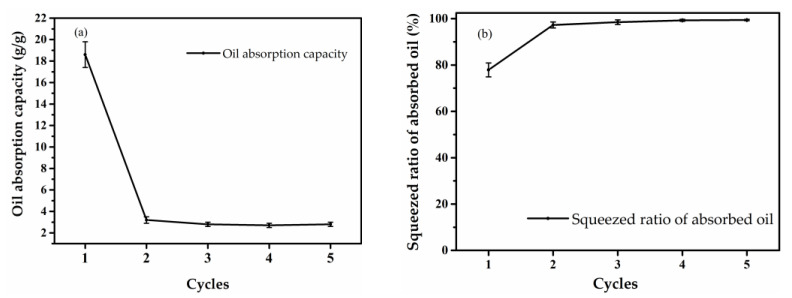
Effect of cycles of sorption on (**a**) oil absorption capacity and (**b**) squeezed ratio of absorbed oil.

The absorbed motor oil can be re-collected through extruding oil containing aerogel. Figure 9a and Table S5 show the effect of cycles of sorption on the oil absorption capacity of the TW01 aerogel on 5W-30 at 25 °C. The sample achieved a high absorption capacity of 18.6 ± 1.2 g/g in cycle 1. However, the absorption capacity obviously dropped to 3.2 ± 0.3, 2.8 ± 0.2, 2.7 ± 0.2 and 2.8 ± 0.2 g/g in cycles 2, 3, 4 and 5, respectively. Figure 9b and Table S5 exhibit the squeezed ratio of the absorbed oil. 77.9 ± 3.0, 97.3 ± 1.3, 98.5 ± 1.0, 99.3 ± 0.4 and 99.4 ± 0.3% of the absorbed motor oil was re-collect after cycles 1, 2, 3, 4 and 5, respectively, by simple squeezing. This phenomenon can be explained based on the change in the aerogel volume and weight. The sample used in cycle 2 was squeezed while collecting the oil absorbed in cycle 1, causing a partial collapse of the porous structure of the aerogel, which could not be completely recovered in a later cycle. In addition, the adsorbed oil could not be completely removed, and the initial weight of the sample in cycle 2 increased, which sharply reduced the oil absorption values. In later cycles, the oil absorption values and the squeezed ratio are similar to the value after the first cycle, which may be due to the sample structure having not changed anymore and the weight of the remaining oil in the squeezed aerogel being close in each cycle.

**Figure 10:**
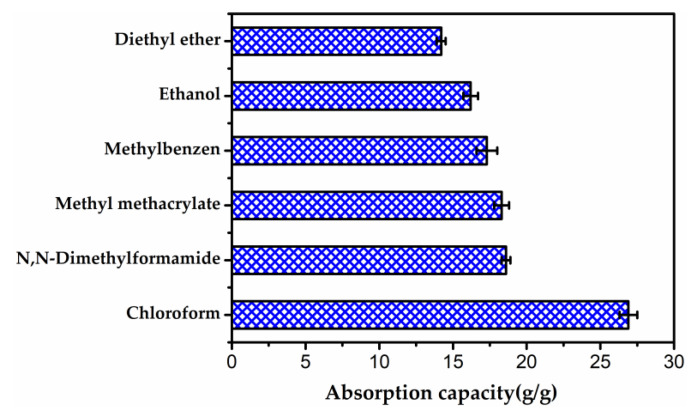
Absorption capacity of TW01 for various solvents.

TW01 was selected to evaluate the absorption capability of aerogels for various organic solvents, which are frequent pollutants and leaks in ordinary routine or in industrial applications. The absorption capacity of TW01 varies from 14.2 ± 0.3 to 26.9 ± 0.6 times its own weight, as illustrated in Figure 10 and Table S6, depending on the physical characteristics of the absorbed organic solvents. The adsorption capability of various previously reported materials for oil and organic solvents is displayed in Table 3. As expressed, the adsorption capability of TWF aerogel is comparable to that of many previously reported adsorbent materials. Even though the sample has a lower absorption capacity than some of the reported materials, the raw material is cheaper and the preparation procedure is more straightforward.

**Figure 11:**
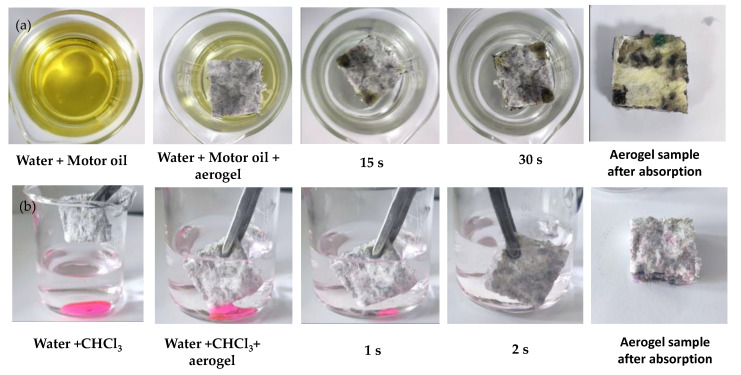
The oil–water separation with TW03 after modified MTMS. (**a**) Sorption process of motor oil 5W-40. (**b**) Sorption process of trichloromethane (dyed by rhodamine B).

To evaluate the oil–water separation performance of the TWF aerogel, light oils and dense oils are prepared to imitate oil/water mixtures. Figure 11 shows the application of TWF aerogel for the removal of oil from water. As shown in Figure 11a hydrophobic TWF aerogel floats on mixtures and rapidly and completely absorbs motor oil 5W-40 within 30 s, which leaves clean water with no visible oil drops. Figure 11b displays the successful removal of trichloromethane (dyed with rhodamine B) from water. Once TWF aerogel contacted the trichloromethane sinking under the water, it absorbed quickly and perfectly as well; these results suggest that this material has excellent oleophilic and hydrophobic properties. Thus, the TWF aerogel can efficiently separate both light oils and dense oils from water. Furthermore, the collection of the absorbed oil is very simple through the crimping of the aerogel.

## 3. Conclusions

In conclusion, a facile and cost-effective method of the textile waste fibers aerogels from textile waste has been successfully developed. After being modified with MTMS, the developed aerogels exhibit excellent hydrophobicity with a water contact angle of up to 136.9° ± 2.3°and oil–water separation performance. Textile waste fibers aerogels can be used as heat insulation materials for buildings with excellent heat insulation properties and thermal stability. It is found that the initial fiber concentration, the temperature and the oil viscosity significantly affect the oil absorption. Due to the TWF aerogel’s low density and high porosity, its maximum absorption value reaches 26.9 ± 0.6 g/g, whereas the absorption capacity decreases obviously after the compression cycle, limiting its reuse of oil absorption. The formation of waste textiles into aerogel is expected to increase the commercial value of textile waste in the future. The application prospects of TWF aerogels may be further expanded if effective strategies are executed to modify TWF aerogels to obtain interactive response characteristics such as temperature, pH, light, electric field, magnetic field, and so on.

## 4. Materials and Methods

### 4.1. Materials

The textile waste fibers (TWF, supplied by Shangyu Textile Co., Ltd., Suzhou, China) have a length of approximately 2–20 mm from crushed waste clothing and are made up of nearly 90% polyester and 10% cotton, two major fiber types on the international market. Polyvinyl alcohol (PVA, degree of polymerization: 1799 ± 50, content ≥ 90.5%), glutaraldehyde (GA, 25% in water), sodium hydroxide (NaOH, 96.0 wt.%) and methyltrimethoxysilane (MTMS, 98 wt.%) were purchased from Sinopharm Chemical Reagent Co., Ltd. (Shanghai, China). Sodium chloride (NaCl, 99 wt.%) and Trichloromethane (CHCl_3_, 99 wt.%) were obtained from Macklin Biochemical Corporation (Shanghai, China). Motor oils of 5w-30 and 5w-40 were purchased from Exxon Mobil Corporation. All the above reagents were used without any purification steps.

### 4.2. Methods

#### 4.2.1. Fabrication of Textile Waste Fibers Aerogels

The textile waste fibers aerogels were developed through using textile waste as the matrix and polyvinyl alcohol (PVA) and glutaraldehyde (GA) as crosslinking agents. The specific processes are as follows and shown in Figure 1. In the initial stages of the process, the TWF were pretreated with alkali NaOH (40 g/L) at 80 °C for 30 min with a 1:100 material-to-liquor ratio before being used as the matrix [11,44]. Second, the different volumes of TWF were immersed in the PVA/GA/H2O mixed solution and next sonicated at 400 W, 80 °C for 20 min. The TWF concentrations were changed from 2 to 5 wt.%, The specific ratio of the TWF and the PVA is shown in Table 1. 50 μL the GA solution was added to each sample. Then the mixture was cured at 80 °C for 3 h in the oven. Lastly, the mixture was put in the refrigerator at −18 °C to gel for 12 h and then freeze-dry at −60 °C for 48 h.

#### 4.2.2. Development of the Hydrophobic TWF Aerogels

The developed hydrophilic TWF aerogels were modified with MTMS on their highly porous network surface to form the hydrophobic aerogels materials [45,57,69]. The TWF aerogels and a small beaker containing MTMS were placed in a large closed container. Then, the container was heated in the oven at 70 °C for 3 h. After the TWF aerogel surface was completely silanized, the excessive MTMS was removed by placing the aerogel sample in a vacuum oven.

#### 4.2.3. Characterization

The structure and morphology of the TWF aerogels were examined using a scanning electron microscope (SEM, Merlin Compact, Carl Zeiss AG, Oberkochen, Germany). The samples were modified with an ultra-thin layer of gold for the 90 s at 20 mA using a sputter coater (SD-900, Vision Precision Instruments, Dongguan, China).

The hydrophobicity of the MTMS-modified aerogels was investigated by the water contact angle, which was carried out on an OCA25 goniometer (Dataphysics Products Inc., Filderstadt, Germany). During the test, 10 μL water drops were dripped onto the surface of the aerogels and controlled by the syringe system of the tester. The contact angle was calculated according to the acquired photographic image with the angle between the drop and surface measurement on the software system.

Nitrogen sorption isotherms with standard Brunauer-Emmett-Teller (BET) analysis were used to calculate specific surface areas. Through the adsorption branch of the isotherms, the pore size (Figure S1) was determined using the Barrett-Joyner-Halenda (BJH) method (Micromeritics ASAP 2460, Micromeritics Instrument Corporation, Norcross, GA, USA). All samples were degassed at 50 °C for 24 h under a vacuum before analysis.

The thermal conductivity was analyzed using the TPS2500S Hot Disk (Hot Disk AB, Uppsala, Sweden) and the transient plane source method. The samples were tested at a room temperature of 25 °C.

The thermal gravimetric analysis (TGA) was carried out using HCT-3 Thermogravimetric Analyzer (Henven Corporation, Beijing, China) to evaluate the thermal stability of the specimens by heating them from room temperature to 800 °C at 10 °C min^−1^ under air.

A universal testing machine (6800, Instron Corporation, Boston, MA, USA) was applied to the compression test, with a constant loading rate of 1 mm/min. The compressive Young’s modulus was elected as the initial (0–10%) linear portion of the stress-strain curve slope.

Every prepared sample is measured and weighted in order to determine its density. The porosity (φ) is calculated using the following formula dependent on the density (ρ_g_) of aerogel and the average density of components (ρ_c_) as indicated by the following.

(1) $$\varphi \;=\left(1-\frac{\rho_{g}}{\rho_{c}}\right)*100\%$$

The aerogel was put into a beaker with motor oil or organic solvents to study the absorptivity, the process displayed on Figure 7. Two motor oil used for the absorption tests were 5W-30 and 5W-40. The motor oil specifications are displayed in Table S4. The sample was weighed and placed in motor oil for 1 h to ensure a swelling equilibrium. Then the wet sample was lifted from the oil container, drained for 30 s in air and weighed again. The motor oil absorption capacity was calculated using Equation (2). The adsorption process of organic reagents is the same as that of motor oil adsorption. The wet sample was squeezed by hand, and it was weighed again. The test was repeated several times. The squeezed ratio of crude oil (*Q_s_*) was calculated using Equation (3).

(2) $$Q_{t}=\frac{M_{w}-M_{d}}{M_{d}}$$

*Q_t_* is the crude oil absorption capacity of the aerogel, *M_w_ (g)* is the weight of the aerogel after absorption, and *M_d_ (g*) is the weight of the aerogel before absorption.

(3) $$Q_{s}=\frac{M_{w}-M_{s}}{M_{w}-M_{d}}*100\%$$

*M_s_ (g)* is the weight of the aerogel after squeezing.

## Figures and Tables

**Table 1:** Chemical compositions of various textile waste fibers aerogels, density, porosity, thermal conductivity and BET surface area.

Sample Name	Composition	Fiber Conc. (wt.%)	Density (g/cm^3^)	Porosity (%)	Thermal Conductivity (W/m·K)	BET Surface Area (m^2^/g)
TWF1a	TWF:PVA = 1	2.0	0.067 ± 0.003	94.8 ± 0.2	0.057 ± 0.002	-
TWF1b	TWF:PVA = 3	2.0	0.045 ± 0.002	96.6 ± 0.2	0.051 ± 0.001	-
TWF1c	TWF:PVA = 4	2.0	0.040 ± 0.002	96.9 ± 0.3	0.049 ± 0.001	-
TW01	TWF:PVA = 2	2.0	0.044 ± 0.002	96.7 ± 0.2	0.052 ± 0.001	12.0
TW02	TWF:PVA = 2	3.0	0.060 ± 0.003	95.4 ± 0.3	0.058 ± 0.002	10.6
TW03	TWF:PVA = 2	4.0	0.068 ± 0.003	94.8 ± 0.2	0.059 ± 0.002	9.0
TW04	TWF:PVA = 2	5.0	0.096 ± 0.005	94.2 ± 0.4	0.061 ± 0.002	7.3

**Table 2:** Thermal conductivity of PVA composite, polyester composite, Cotton composite aerogel and textile waste fibers composite.

Materials	Thermal Conductivity (W/m·k)	Reference
TWF/PVA Aerogel	0.049–0.062	This work
Air	0.026	[47]
PVA plastic	0.200	[48]
Pristine PVA Aerogel	0.0408	[49]
PET Fiber	0.15	[41]
PET Plastic	0.20	[50]
Cotton	0.047	[51]
Cotton/Cellulose Aerogel	0.044–0.055	[52]
PVA/TA/SA Aerogel	0.043–0.060	[40]
PVA/GA/CNF Aerogel	0.044–0.067	[53]
Waste Tissue Paper/PVA Aerogel	0.098–0.120	[54]
Wool Waste Fibers Aerogel	0.049–0.060	[55]
Wood Fibers/Textile Waste Fibers Aerogel	0.078–0.089	[56]

**Table 3:** Performance comparison of the reported sorbent materials.

Sorbent Material	Absorbed Substances	Sorption Capacity (g/g)	Reference
TWF/PVA Aerogel	oils and organic solvents	14–27	This work
BN/PVA Aerogel	CCl_4_ and n-hexane	12–38	[59]
Chitosan/Silica Composite Aerogel	oils and organic solvents	13–30	[60]
carbon nanotubes/PVA Aerogels	oils and organic solvents	17–39	[61]
MOF/Carbon Nanotubes/Cotton Aerogels	oils and organic solvents	48–84	[62]
MOF-Coated Cotton Fiber Composite	oils and organic solvents	25–48	[63]
Wood /PVA Sponge	oils and organic solvents	4–27	[64]
Cellulose-Based Aerogel	oils and organic solvents	42–99	[65]
Polyimide/MXene Aerogels	oils and organic solvents	18–58	[66]
PLA/ lignocellulosic Aerogels	oils and organic solvents	28–70	[67]
Aramid Nanofibers/PVA aerogel	oils and organic solvents	32–65	[68]

## Data Availability

Not applicable.

## Supplementary Materials

The following supporting information can be downloaded at: https://www.mdpi.com/article/10.3390/gels8100684/s1, Table S1: Thermal stability of aerogels; Table S2: The mechanical property of the TWF aerogels; Table S3:The motor oil absorption capabilities of TWF aerogels; Table S4: Specification of motor oil; Table S5: Effect of cycles of sorption on oil absorption capacity and squeezed ratio of absorbed oil; Table S6: Absorption capacity of TW01 for various solvents; Figure S1: Pore size distribution: (a) TW01, (b) TW02, (c) TW03 and (d) TW04.
