# Supplementary material for: Fabrication of Textile Waste Fibers Aerogels with Excellent Oil/Organic Solvent Adsorption and Thermal Properties
Source: Gels. 2022 Oct 21;8(10):684. doi: 10.3390/gels8100684 (PMC9601950; doi:10.3390/gels8100684)
Supplement: Supplementary file 1 [file gels-08-00684-s001.zip › gels-1974537-supplementary.pdf]

gels

Supplementary Material

Fabrication of Textile Waste Fibers Aerogels with Excellent Oil/Organic Solvent Adsorption and Thermal Properties

Chunlei Dong[1], Yangzhao Hu[1], Yuxuan Zhu[1], Jiale Wang[1], Xuerui Jia[1], Jianbing Chen[1,2,*], Jingliang Li[2*]

Table S1 Thermal stability of aerogels

Sample Name	125 °C weight loss (%)	$T_{5\%}$ [1](°C)	$T_{10\%}$ [2] (°C)
Uncoated aerogel	4.5	233.6	270.2
TW01	0.8	263.2	279.1
TW02	1.1	255.9	288.9
TW03	0.4	270.3	292.8

[1] The temperature of weight loss 5%.
[2] The temperature of weight loss 10%.

Table S2. The mechanical property of the TWF aerogels.

Sample Name	Young's modulus（kPa）	Strain 10%（kPa）	Strain 50%（kPa）
TW01	1.30 ± 0.16	19.9	60.1
TW02	2.97 ± 0.21	42.1	128.6
TW03	4.34 ± 0.19	54.0	199.6
TW04	5.24 ± 0.22	67.1	235.1

Table S3. The motor oil absorption capabilities of TWF aerogels.

Sample Name	Oil absorption capacity (g/g)			
	5W-30		5W-40	
	25 °C	75 °C	25 °C	75 °C
TW01	18.6 ± 1.2	15.3 ±0.3	16.0 ± 1.5	15.5 ± 0.3
TW02	14.1 ± 0.5	13.7 ± 0.3	13.5 ± 0.5	13.3 ± 0.3
TW03	11.6 ± 0.5	10.2 ± 0.3	10.6 ± 0.4	10.1 ± 0.2
TW04	9.4 ± 0.2	8.3 ± 0.1	8.7 ± 0.3	8.4 ± 0.1

Citation: Dong, C.; Hu, Y.; Zhu, Y.; Wang, J.; Jia, X.; Chen, J.; Li, J. Fabrication of Textile Waste Fibers Aerogels with Excellent Oil/Organic Solvent Adsorption and Thermal Properties. *Gels* **2022**, *8*, 684. https://doi.org/10.3390/gels8100684

Academic Editor: Miguel Sanchez-Soto

Received: 8 October 2022
Accepted: 19 October 2022
Published: 21 October 2022

Publisher's Note: MDPI stays neutral with regard to jurisdictional claims in published maps and institutional affiliations.

Copyright: © 2022 by the authors. Licensee MDPI, Basel, Switzerland. This article is an open access article distributed under the terms and conditions of the Creative Commons Attribution (CC BY) license (https://creativecommons.org/licenses/by/4.0/).

Table S4. Specification of motor oil.

Motor Oil	Viscosity (mPa·s)		
	**25 °C**	**50 °C**	**75 °C**
5W-30	96.6	50.4	21.0
5W-40	117.8	55.6	29.6

Table S5. Effect of cycles of sorption on oil absorption capacity and squeezed ratio of absorbed oil.

cycles	Oil absorption capacity(g/g)	Squeezed ratio of absorbed oil (%)
1	18.6 ± 1.2	79.9 ± 3.0
2	3.2 ± 0.3	97.3 ± 1.3
3	2.8 ± 0.2	98.5 ± 1.0
4	2.7 ± 0.2	99.3 ± 0.4

5	2.8 ± 0.2	99.4 ± 0.3

Table S6. Absorption capacity of TW01 for various solvents

Organic Solvent	Density（g/cm^3）	Absorption capacity(g/g)
Chloroform	1.490	26.9 ± 0.6
N,N-Dimethylformamide	0.944	18.6 ± 0.3
Methyl methacrylate	0.943	18.3 ± 0.5
Methylbenzen	0.872	17.3 ± 0.7
Ethanol	0.789	16.2 ± 0.5
Diethyl ether	0.713	14.2 ± 0.3

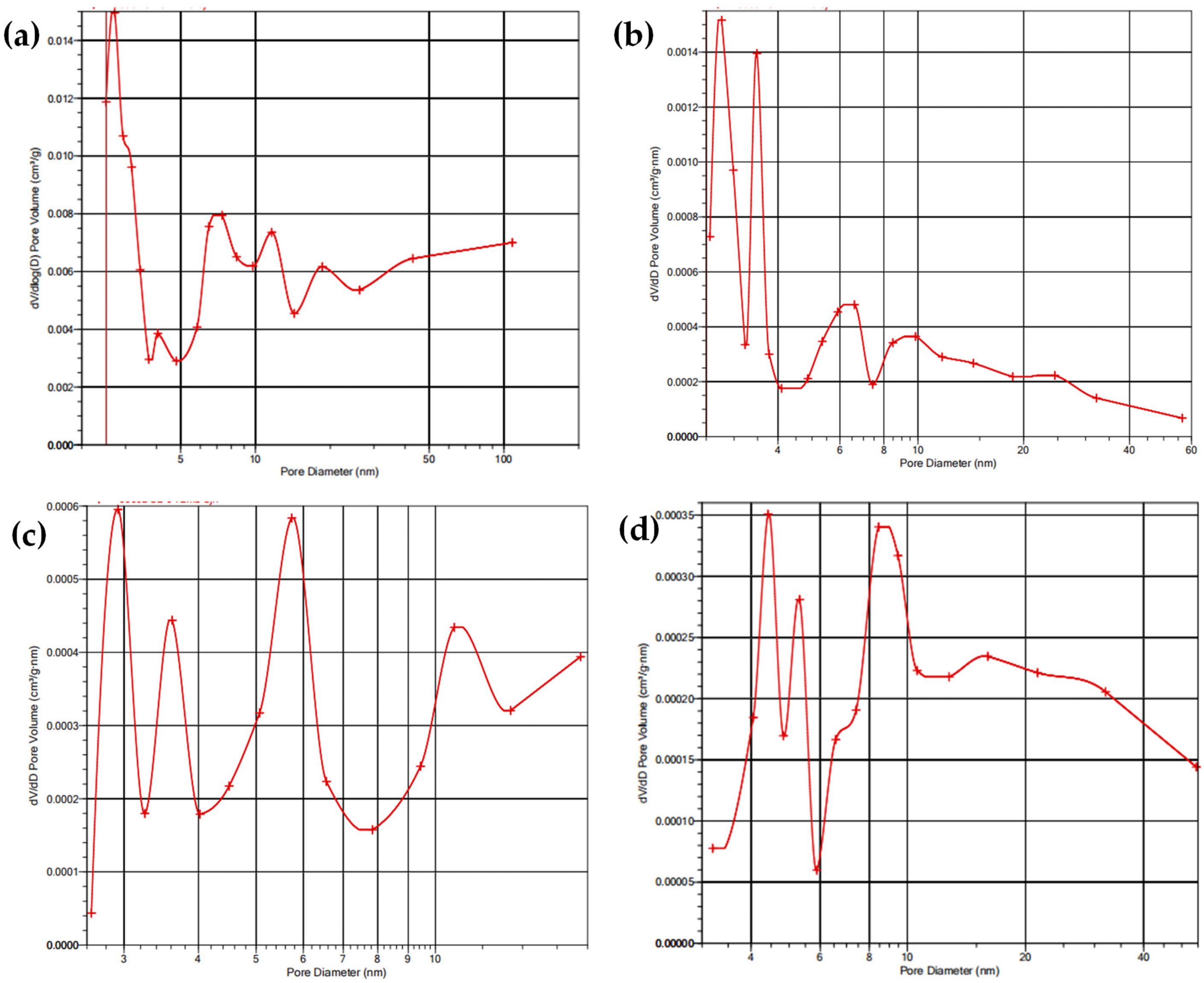

Figure S1. Pore size distribution: a) TW01, b) TW02, c) TW03 and d) TW04.
